# Community Composition and Structure Affect Ecosystem and Canopy Water Use Efficiency Across Three Typical Alpine Ecosystems

**DOI:** 10.3389/fpls.2021.771424

**Published:** 2022-01-20

**Authors:** Yuzhe Li, Xinyuan Zhang, Quanqin Shao, Jiangwen Fan, Zhi Chen, Jinwei Dong, Zhongmin Hu, Yue Zhan

**Affiliations:** ^1^Key Laboratory of Land Surface Pattern and Simulation, Institute of Geographical Sciences and Natural Resources Research, Chinese Academy of Sciences, Beijing, China; ^2^School of Earth Sciences and Resources, China University of Geosciences, Beijing, China; ^3^Key Laboratory of Ecosystem Network Observation and Modeling, Synthesis Research Center of Chinese Ecosystem Research Network, Institute of Geographic Sciences and Natural Resources Research, Chinese Academy of Sciences, Beijing, China; ^4^School of Geography, South China Normal University, Guangzhou, China; ^5^College of Forestry, Nanjing Forestry University, Nanjing, China

**Keywords:** evapotranspiration partition, ecosystem water use efficiency, canopy water use efficiency, Qinghai–Tibetan Plateau, alpine ecosystem, community characteristics

## Abstract

Unique ecosystems distributed in alpine areas of the Qinghai–Tibetan Plateau play important roles in climate change mitigation, local food supply, and conservation of species diversity. To understand the water use efficiency (WUE) of this fragile and sensitive region, this study combined observed data from the eddy covariance system and the Shuttleworth–Wallace (S-W) model to measure the continuous mass exchange, including gross primary productivity (GPP), evapotranspiration (ET), and canopy transpiration (T) throughout 2 or 3 years (2016–2018) in three common alpine ecosystems (i.e., alpine steppe, alpine meadow, and alpine swamp). These ecosystems represent a water availability gradient and thus provide the opportunity to quantify environmental and biological controls on WUE at various spatiotemporal scales. We analyzed the ecosystem WUE (WUEe; defined as the ratio of GPP to ET) and canopy WUE (WUEc; defined as the ratio of GPP and canopy T). It was found that the yearly WUEe was 1.40, 1.63, and 2.16 g C kg^–1^ H_2_O, and the yearly WUEc was 8.93, 2.46, and 5.19 g C kg^–1^ H_2_O in the three typical ecosystems, respectively. The controlling factors of yearly WUE diverged between WUEe and WUEc. We found that plant functional group proportion (e.g., gramineous and Cyperaceae) highly explained the yearly WUEe variation across sites, and a good correlation was observed between community species diversity and WUEc. These findings suggest that community composition and trait change are critical in regulating WUEe and WUEc across different alpine ecosystems and that the regulation mechanisms may differ fundamentally between WUEe and WUEc.

## Introduction

The Qinghai–Tibetan Plateau has received international attention because of its contribution to the global carbon budget and the sensitivity and fragility of the alpine ecosystems. This region is considered one of the world’s most sensitive areas to climate change (Liu D. et al., 2018; Liu S. et al., 2018). The Three Rivers Headwaters Region, located in the central of Qinghai–Tibetan Plateau, is the most critical area for reserving water resources and maintaining alpine biodiversity (Liu et al., 2008; Wang et al., 2019), and is also an important pasture for the local free-grazing livestock production (Zhang L.X. et al., 2014; Zhang et al., 2019). This area has already experienced a considerable climate change, including air temperature increase (0.16°C decade^–1^) and precipitation change (Wang et al., 2008). These changes may profoundly affect local carbon and water cycles, thus influencing the sustainable use of regional limited water, forage resources, and the welfare of the local people.

Water use efficiency (WUE), the ratio of water loss to carbon gain, links water and carbon cycling together (Hu et al., 2008; Keenan et al., 2013; Ali et al., 2017) and is an important and inherent ecosystem characteristic related to ecosystem productivity and water budget (Ponton et al., 2006; Zhou et al., 2018). WUE change may greatly impact on both forage productivity and regional water balance. Thus, a more accurate and deeper understanding of the dynamics of WUE at various observation scales is conducive to the verification and improvement of the accuracy of assessment of regional resource use (Hu et al., 2008; Niu et al., 2008) and the prediction of the climate effect on the limited forage supply and water resource change.

Ecosystem-scale WUE (WUEe) is commonly defined as the ratio between gross primary productivity (GPP) and evapotranspiration (ET) (Law et al., 2008), whereas canopy-scale WUE (WUEc) is defined as the ratio between GPP and canopy transpiration (T) (Niu et al., 2008, 2011). Owing to various analysis scales and data collection techniques, there are differences in the calculation of “productivity” and “water loss” in previous research on WUE and its controlling factors (Zhou et al., 2018; Liu R. et al., 2021). Previous studies have indicated that the variation and composition of ecosystem vapor losses play an important role in shaping WUE and regional environmental conditions of ecologically fragile areas (Bremer et al., 2001; Hu et al., 2009, 2010). However, water resource management based on ET measurements may overestimate the actual water losses of communities, resulting in low WUE (Li et al., 2016). Accordingly, it is necessary to separate the composition of ET accurately and reliably when investigating WUEe, WUEc, and their controlling mechanisms. Measurements, such as manipulative measurement with portable infrared analyzer (Ens et al., 2015; Li et al., 2016), stable isotopes (Williams et al., 2004; Hu et al., 2014), lysimeter (Gebler et al., 2015), and eddy covariance techniques (Li et al., 2019b), are used to estimate ET and its components. Eddy covariance systems are feasible methods of continuous observation of ET (Ponton et al., 2006; Li et al., 2016), but are weak in partitioning ET components. The Shuttleworth–Wallace (S-W) model (Shuttleworth and Wallace, 2010), characterized by its strength in partitioning ecosystem ET components, has been widely used for extracting T from ET since its publication (Hu et al., 2008; Li et al., 2015). Using the data obtained for the eddy covariance system as input, the measured ET can be divided into T and E at a half-hourly resolution, which might resolve the difficulty of continuous ET observation with partitioning.

Understanding the mechanisms underlying variation in WUE response at different scales is necessary to provide data for the validation of any upscaling method (Wu et al., 2015; Liu D. et al., 2018), but there are still biases and conflicts of understanding in WUE controlling mechanisms. In previous studies, WUEe was commonly predicted to decrease under climate warming (Bell et al., 2015), and decrease (Huxman et al., 2004; Scanlon and Albertson, 2010) or no change (Lauenroth et al., 2000) with increasing precipitation. Leaf area index (LAI) and carbon assimilation are positively related to WUEe (Farquhar et al., 1989; Hu et al., 2008). In canopy scale, WUEc suspected depends on the responses of both plant physiology and community structure (Niu et al., 2011). The plant community is usually constituted with many coexisting species that differ in water use strategies that adapt the various ecological niches and the changing environment (Ens et al., 2015; Li et al., 2019a), thereby promoting stability of community and ecosystem productivity. Correspondingly, it can be inferred that changes in community traits, especially species composition, may fundamentally affect WUE (De Boeck et al., 2006). Nevertheless, few attempts (Niu et al., 2011; Li et al., 2016) have been made to compare WUE response, especially WUEc to community structure and composition change. In the Qinghai–Tibetan Plateau, the mountain upheaval has caused a distinct elevation gradient and blocking effect, producing a drastic variation in species composition and a varied community structure. To the best of our knowledge, no attempt has been made to analyze the relationships between community traits and different scales of WUE in this region. Reducing this uncertainty would help local pasture and water resources management in accurate predictions and adaptation to climate change.

The overall objective of this study was to quantify the seasonal and site-to-site variations in the WUEe and WUEc in three typical Qinghai–Tibetan Plateau ecosystems (i.e., alpine steppe, alpine meadow, and alpine swamp). In particular, we were interested in the underlying regulation mechanism of the less-reported WUEc and the relationship between alpine community traits variation and WUE. The specific objectives of our study were as follows: (1) to compare the carbon assimilation and ET components, and WUEe and WUEc among the three alpine ecosystems; (2) to improve the current understanding of driving factors on seasonal variation of WUEe and WUEc in alpine ecosystems; and (3) to test our hypothesis that the community composition plays a special role in regulating WUE across alpine ecosystems.

## Materials and Methods

### Study Sites

**FIGURE 1:**
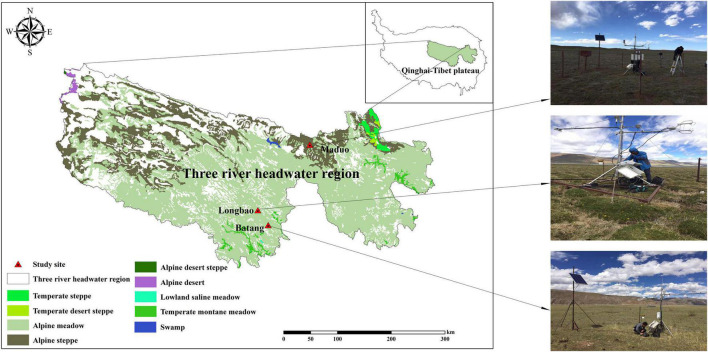
Grassland types and location of the three sites in the Three River Headwater Region, Qinghai–Tibetan Plateau.

Observation and field survey were conducted at three alpine sites located in the Qinghai–Tibetan Plateau. The community of these sites represents the most prevalent vegetation types of the Qinghai–Tibetan Plateau and exhibits a clear water availability gradient (Figure 1). They include an alpine steppe located in Maduo County, dominated by *Stipa purpurea*, the soil of this community is frigid calcic soil; an alpine meadow located in Batang, Yushu County, dominated by *Kobresia humilis*, the soil of this community is felty soil; and an alpine swamp located in Longbao Nature Reserve, Yushu County, dominated by *Kobresia littledalei*, the soil of this community is bog soil. The land use of the three sites was free grazing and all the sites are located in Plateau mountain climate areas. The three sites were fully instrumented to enable the state-of-the-art measurements of vapor and CO_2_ fluxes. The geographical location, elevation, dominant species, and soil physicochemical properties of the three sites are listed in Table 1.

**TABLE 1:** Site information and community characteristics of the three grassland ecosystems on the Qinghai–Tibetan Plateau.

Site	Alpine steppe	Alpine meadow	Alpine swamp
Location	35°6′12′′N, 97°58′40′′E	32°50′49′′N, 96°57′3′′E	33°12′14′′N, 96°33′7′′E
Elevation (m)	4316	3980	4220
Mean precipitation (mm)	392.99	578.78	573.73
Mean temperature(°C)	−3.36	2.12	0.74
Soil type	Frigid calcic soils	Felty soils	Bog soils
Dominant species	*Stipa purpurea*	*Kobresia humilis*	*Kobresia littledalei*
Cover (%)	66.67 ± 3.33a	99.33 ± 0.67b	100.00 ± 0.00b
Height (cm)	20.3 ± 0.57a	46.00 ± 12.17b	21.67 ± 1.67a
Maximum LAI (m^2^ m^–2^)	0.6	2.8	2.5
Aboveground biomass (g m^–2^)	66.29 ± 11.22a	275.92 ± 25.36b	313.28 ± 25.41b
Total biomass (kg m^–2^)	2.00 ± 0.23a	3.75 ± 0.30b	19.88 ± 4.12c
Gramineous biomass proportion (%)	62.41 ± 12.50a	42.08 ± 12.32b	0.00 ± 0.00c
Cyperaceae biomass proportion (%)	20.55 ± 9.96a	17.96 ± 9.65a	83.00 ± 6.74b
Specie richness	5.33 ± 0.67a	15.33 ± 0.88b	10.67 ± 0.67c
Shannon–Wiener index	1.31 ± 0.11a	2.52 ± 0.06b	2.02 ± 0.18c

*Mean ± SD; different letters in the same row indicate significant difference at p < 0.05.*

### Observation of Fluxes, Meteorological Parameters, and Leaf Area Index

The device configuration was kept consistent across the three sites; each observation site had a 4-m high micrometeorological observation tower, with the open eddy covariance system (OPEC) installed at 2.5 m height. The OPEC system is mainly used for observation of turbulent flux and includes a three-dimensional ultrasonic anemometer (CSAT3; Campbell, CA, United States) and infrared gas analyzer (EC150; Campbell, CA, United States) to determine the three-dimensional wind speed and the density of CO_2_ and H_2_O in the atmosphere. The data collector (CR3000; Campbell, CA, United States) had a sampling frequency of 10 Hz and was responsible for the collection and calculation of the average flux of 30 min for storage. Observation equipment of the aboveground micrometeorological elements includes NR01 four-component net radiation sensor, HMP155a temperature and humidity sensor, and NR-LITE net radiation sensor. The soil water content (SWC) was recorded using an underground CS616 soil moisture sensor, and the soil temperature was recorded using a 109SS soil temperature sensor.

ChinaFLUX has developed a series of methods for processing flux data including flux calculation, gap-filling, partitioning, and quality assurance methods (Yu et al., 2006; Han et al., 2020). In this study, the flux data were filtered and corrected using the standard ChinaFLUX process, which involves coordinate rotation, Webb–Pearman–Leuning correction (Webb et al., 2010), outlier filtering, nighttime CO_2_ flux correction using site-specific thresholds of friction velocity (u*), and gap-filling (Reichstein et al., 2005). The net ecosystem productivity (NEP) values were obtained based on the fully interpolated 30-min data. NEP was partitioned into respiration (Re) and GPP using the functional relationship between nighttime fluxes and soil temperature. The computations were performed using MATLAB software (Math Works Inc., Natick, MA, United States).

At each site, although LAI was measured by harvesting the aboveground biomass at the peak of the growing season (August), these data were not continuous. To configure the integrated LAI dataset with relatively fine-time resolution, normalized difference vegetation index (NDVI) data (8-day average at 1-km resolution^1^) were adopted for the estimation of LAI.

### Evapotranspiration Partitioning and the Shuttleworth–Wallace Model

The S-W model, developed based on the Penman–Monteith model, is widely used in the study of ET in ecosystems, such as farmland, grassland, and forest. This model considers that the latent heat flux from E and the T are two independent sources. In this model, methods for estimating soil surface resistance (*R*_*SS*_) and canopy resistance (*R*_*C*_) are usually study specific. In this study, *R*_*SS*_ was calculated with an empirical function of surface SWC (Mahfouf and Noilhan, 2015) as follows:


(1) $$R_{S\,S}=b_{1}\,{\left(\frac{\theta_{\text{S}}}{\theta }\right)}^{b_{2}}+b_{3}$$

where θ is the average volumetric soil moisture content between 0 and 10 cm, *θ_*S*_* is the saturated water content in the surface soil, and *b*_1_, *b*_2_, and *b*_3_ are empirical constants. *R*_*C*_ was calculated with the modified Ball–Berry model (Ball et al., 1987) as follows:


(2) $$R_{C}=\frac{1}{g_{0}+a_{1}\,\left(\frac{\theta -\theta_{w}}{\theta_{f}-\theta_{w}}\right)\,P_{n}\,h_{s}/C_{s}}$$

where *g*_0_ and *a*_1_ are empirical parameters; θ_*f*_ and θ_*w*_ are ground soil capacity and wilting points, respectively; *P*_*n*_ is the photosynthetic rate; *h*_*s*_ is the leaf surface relative humidity; and *C*_*s*_ is the leaf surface CO_2_ concentration.

In Equations 1, 2, five parameters, namely, *b*_1_, *b*_2_, *b*_3_, *g*_0_, and *a*_1_, are estimated using the Monte Carlo method (Hu et al., 2009), and the estimation method of other resistances (i.e., air resistance from blade surface to canopy, air resistance from soil surface to canopy, and air resistance from canopy to reference height) is consistent with Shuttleworth and Wallace (2010).

### Survey of Community Characteristics

Every year, at the peak of the growing season (August), a randomized quadrat method with a total of 15 survey quadrats (1 m × 1 m) was applied in each site. At each site, we randomly installed 5 replicate biomass harvest quadrats and 10 species survey quadrats. The percentage cover of each species and the entire vegetation cover were visually estimated in the biomass harvest quadrats. In addition, the harvest quadrats were used for destructive sampling to determine the community characteristics, including aboveground and belowground biomass, and soil properties. The aboveground biomass of each species in each destructive sampling plot was clipped to ground level and the living fraction was separated. The 0- to 30-cm belowground biomass was sampled using the root drill method, as described by Fan et al. (2008). All the aboveground biomass and belowground biomass were oven-dried at 70°C for 48 h and then weighed. In each species survey quadrat, the species composition, percentage cover, and average height of each species were recorded.

The following species diversity indices were calculated as follows:

(1) Species richness, *S*, was calculated as the total number of species appeared in observation site; and
(2) The Shannon–Wiener index (*H*′) (Shannon, 1948) was calculated as follows:

(3) $$H^{\prime }=-\sum P\,i\,\text{ln}\,P\,i$$

where *Pi* is the importance value (*IV*) of species *i*; and *IV* (Li et al., 2019a) was calculated as follows:


(4) IV=(relativeheight+relativecoverage+relativefrequency)/3

### Ecosystem and Canopy Water Use Efficiency Calculation

In this study, the WUEe was defined as the ratio between GPP and ET (Li et al., 2016) as follows:


(5) $${\text{WUE}}_{e}=\frac{G\,P\,P}{E\,T}$$

The WUEc was defined as the ratio of GPP to T (Niu et al., 2011) as follows:


(6) $${\text{WUE}}_{e}=\frac{G\,P\,P}{T}$$

where ET is obtained by the eddy covariance system, *T* is obtained by the S-W model, and GPP is obtained as follows (Li et al., 2019b):


(7) GPP=-NEP+Re

The NEP can be obtained from the eddy covariance system as nighttime ecosystem Re was observed by the eddy covariance system and daytime Re was calculated using the functional relationship between nighttime fluxes and soil temperature (Reichstein et al., 2005).

### Statistical Analysis

One-way ANOVA with Tukey’s honestly significant difference (HSD) test was used to analyze the differences in the community characteristics of the three alpine sites. The paired *t*-test was used to compare the seasonal dynamics of ecosystem H_2_O and CO_2_ exchange as well as WUEc and WUEe of the three sites. Linear regression analysis and root mean squard error (RMSE) were applied to examine the consistency between ET measured by the eddy covariance system and that modeled by the S-W model. Linear regression analysis was used to evaluate the relationships between T/ET, LAI, GPP, ET, T, ecosystem microclimate factors, and WUEe or WUEc. Exponential regression analysis was applied to evaluate the relationships between T/ET, LAI, and WUEc. All statistical analyses were performed using SPSS version 12.0 (IBM, Armonk, NY, United States).

## Results

### Microclimate and Community Characteristics at the Three Sites

**FIGURE 2:**
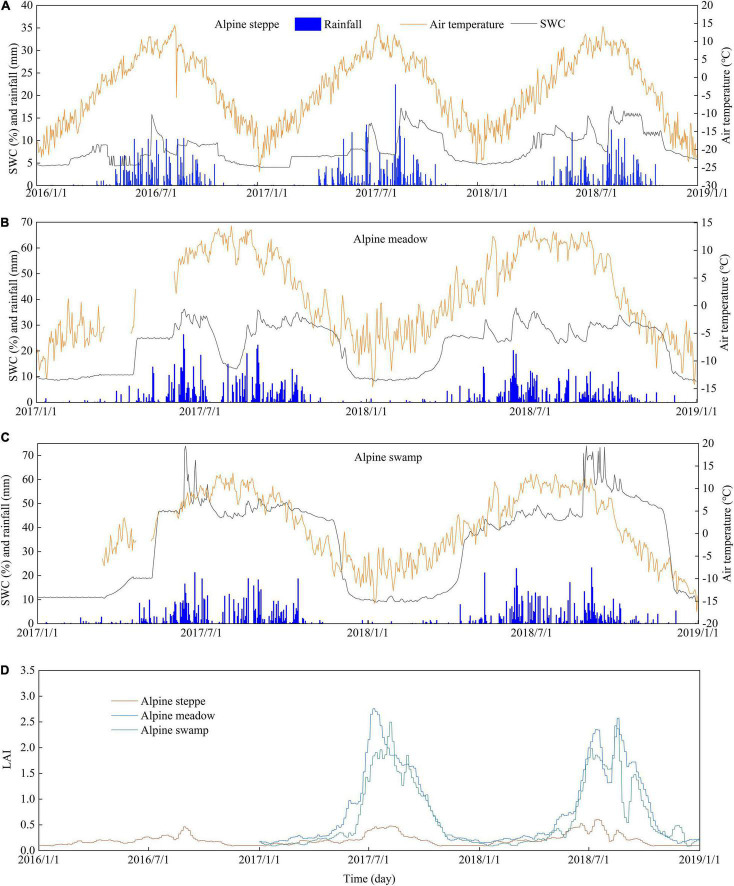
Daily time series of air temperature, rainfall, and soil water content at 20-cm depth at the **(A)** alpine steppe, **(B)** alpine meadow, and **(C)** alpine swamp sites, and **(D)** leaf area index (LAI) at the alpine steppe, alpine meadow, and alpine swamp sites. Note that the *y*-axis scales vary among panels for better presentation.

Seasonal air temperature at the three ecosystems showed a similar one-peak pattern. Both the maximum and minimum air temperatures occurred at the alpine steppe site that is located at a higher altitude (Figure 2). The SWC at the alpine swamp site remained relatively high throughout the growing seasons, whereas SWC at the alpine steppe site was more variable. The rainfall at the alpine steppe site was much lower than that at the other two sites. The maximum LAI commonly occurred in July (Figure 2D).

The community structure and species composition were clearly different between sites. The dominant species is *S*. *purpurea* at the alpine steppe site, and its gramineous biomass portion (about 62.41%) was consistently significantly higher than that of alpine meadow and alpine swamp sites (*p* < 0.05), for which the dominant species were the Cyperaceae family (*K. humilis* and *K. littledalei*, respectively). The species diversity was highest in the alpine meadow with *H*′ reaching 2.52. Similar to *H*′, the species richness was low in the alpine steppe (Table 1).

### Seasonal Variations in Gross Primary Productivity and the Components of Evapotranspiration at the Three Sites

**FIGURE 3:**
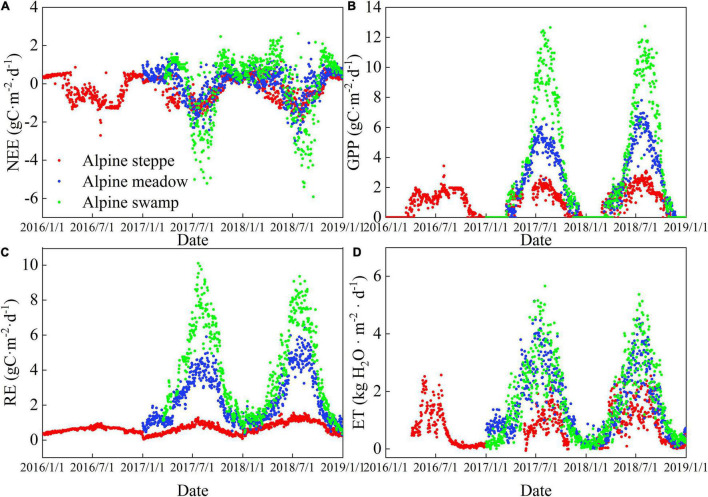
Daily time series of **(A)** net ecosystem exchange, **(B)** gross primary productivity, **(C)** respiration, and **(D)** evapotranspiration (ET) at the alpine steppe, alpine meadow, and alpine swamp sites.

Seasonal variations in net ecosystem exchange (NEE), Re, GPP, and ET at the three sites are shown in Figure 3. NEE refers to the change in ecosystem carbon storage, which mainly reflects the carbon budget of the ecosystem from the perspective of flux observation and has the same physical significance as NEP under certain assumptions. The difference between NEP and NEE is that positive NEP indicates a carbon absorption ecosystem, while when NEE is negative, it indicates the presence of a carbon absorption process. In this study, the NEE showed an apparent U-shaped curve, low in the active periods and high in the early and the late growing season (Figure 3A). However, NEE varied with two or more peaks during each growing season at alpine steppe, and is likely to be caused by summer drought, which is supported by rain and SWC records. The Re typically showed a one-peak seasonal pattern, with the peak occurring in July (Figure 3C). The GPP showed a one-peak pattern similar to Re at the three alpine sites, except for the alpine steppe in 2016 (Figure 3C). The ET showed similar patterns to GPP at the three ecosystems (Figure 3D), suggesting a coupling between carbon and vapor flux.

### Seasonal Variations in Canopy Transpiration/Evapotranspiration and Canopy Transpiration Simulated With the Shuttleworth–Wallace Model at the Three Sites

**FIGURE 4:**
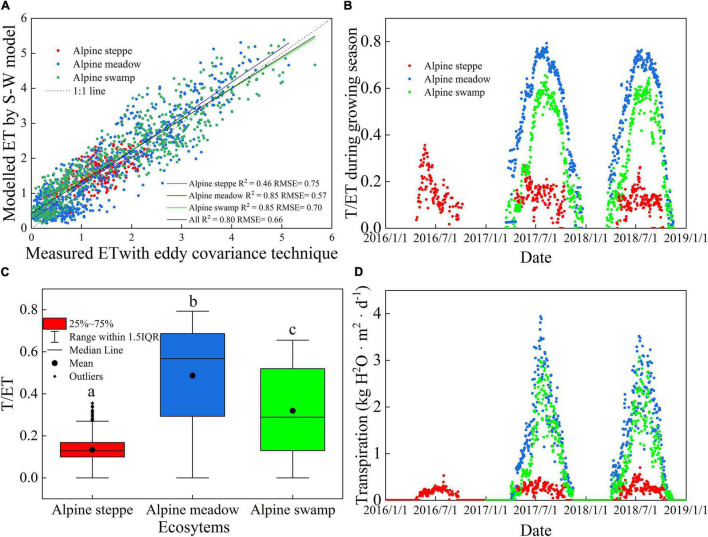
Comparison of **(A)** measured and modeled daily ET, **(B)** daily time series of modeled ratio of transpiration to ET (transpiration [T]/ET), **(C)** the range of daily T/ET, and **(D)** T at the three sites. Different small letters indicate significant differences (*P* < 0.05).

As shown in Figure 4A, the S-W model could successfully estimate vapor losses for the alpine ecosystems (all *R*^2^ = 0.80, RMSE = 0.66), and the RMSE was slightly lower in the alpine meadow. This suggests that the model performs best in mild ecosystems and acceptably in dry steppe and humid swamps. The modeled and measured ET was simulated at the three sites, and the fitting curves were mostly distributed near the 1:1 straight line (Figure 4A). Although the *R*^2^ values varied among the sites, when taking the three sites as a whole, the change of measured ET explained 80% of the modeled ET, suggesting that the S-W model can be successfully applied in most of the alpine ecosystems.

The seasonal dynamics of T/ET and the comparison of daily T/ET are shown in Figures 4B,C, respectively. Seasonal variation of T/ET was one-peak pattern at the three alpine sites. The multiyear average daily T/ET values were 0.13, 0.49, and 0.32 in alpine steppe, alpine meadow, and alpine swamp, respectively (Figure 4C). The T showed one-peak pattern at the three alpine sites (Figure 4D).

### Comparison of Ecosystem Water Use Efficiency and Canopy Water Use Efficiency at the Three Sites

**FIGURE 5:**
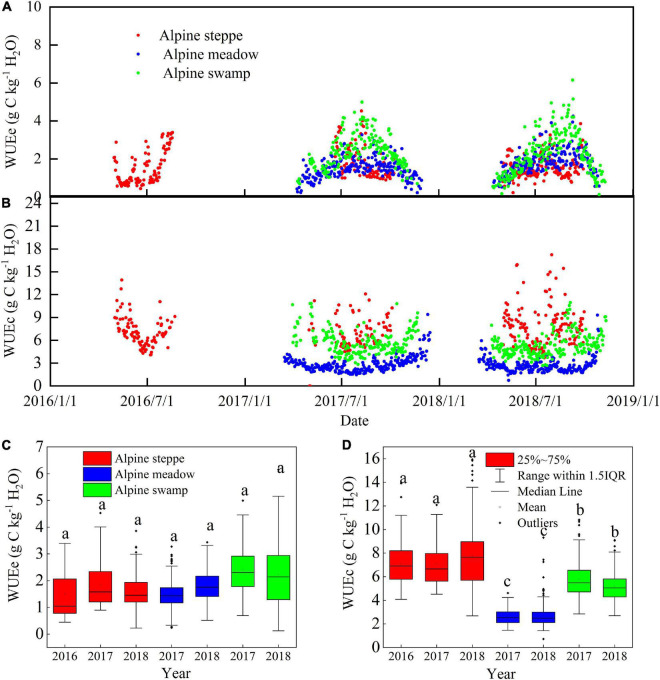
Comparison of seasonal dynamics of **(A)** WUEc and **(B)** WUEe, and the range of daily **(C)** WUEc and **(D)** WUEe in alpine steppe, alpine meadow, and alpine swamp during the study period. Different small letters indicate significant differences (*P* < 0.05).

For all ecosystems, WUEc was lower in the peak growing periods and higher in the early and late growing season (Figure 5B), which is almost opposite to the WUEe trends (Figure 5A). However, the trends of alpine steppe were much less consistent than those of the other two ecosystems, which may be attributed to the harsh and arid environmental conditions that frequently cause drought and frost damage to the vegetation.

The mean daily WUEc and WUEe variation values between years were relatively low at a single site (*p* > 0.05; Figures 5C,D); however, the WUEc values were significantly different between the different sites (*p* < 0.05), while the WUEe values were not (*p* < 0.05). The mean daily WUEc values were 7.28, 2.76, and 5.75 g C kg^–1^ H_2_O in alpine steppe, alpine meadow, and alpine swamp, respectively. The mean daily WUEe values were 1.65, 1.61, and 2.24 g C kg^–1^ H_2_O in these three ecosystems, respectively.

### Daily Variations in Water Use Efficiency and Its Driving Factors

**FIGURE 6:**
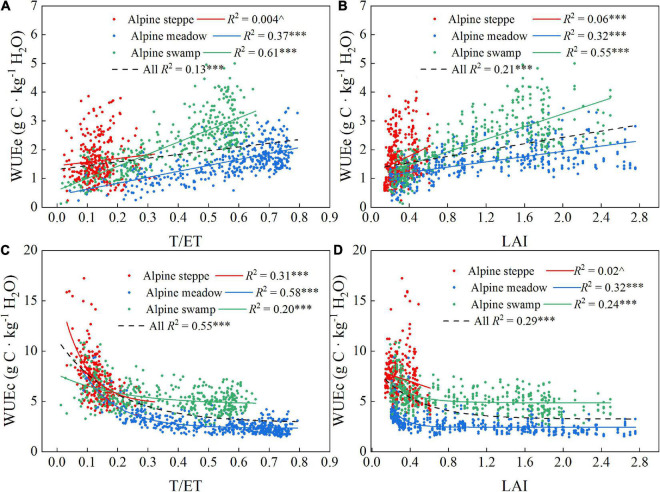
Correlations between T/ET, LAI to WUEe **(A,B)** and T/ET, and LAI to WUEc **(C,D)** of the three alpine ecosystems. *R*^2^ and probabilities of regressions are shown for each correlation. ^*p* > 0.05; ^***^p < 0.001.

The T/ET and LAI values were often significantly correlated with the WUEe and WUEc values (Figure 6); WUEe exhibited positive linear relations with T/ET or LAI in each of the three ecosystems and all three together, whereas WUEc exhibited exponential relations with T/ET or LAI in alpine meadow, alpine swamp, and all three ecosystems together. However, the alpine steppe was a unique case that showed lower *R*^2^, which might be attributed to the frequent meteorological disturbance that restricted the growth of local vegetation in the alpine steppe area.

### Inter-Annual and Inter-Ecosystem Variations in Water Use Efficiency and Its Driving Factors

**FIGURE 7:**
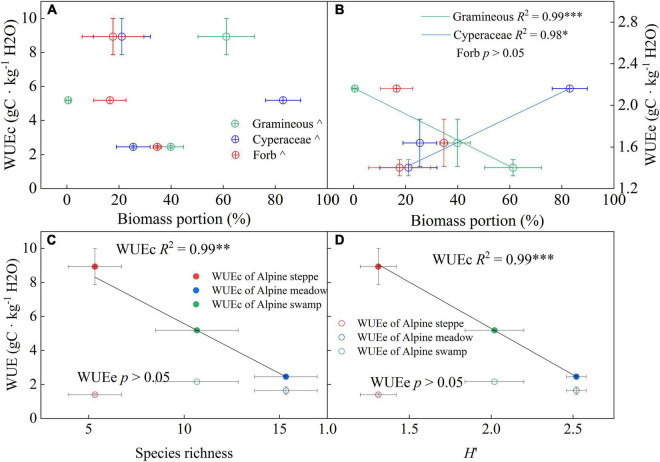
Relationships between yearly WUEe and WUEc and **(A)** total biomass, **(B)** gramineous biomass proportion, and species diversity indices **(C)** species richness and **(D)** Shannon–Wiener diversity index (*H*′) across the three alpine ecosystems. *R*^2^ and probabilities of the regressions are shown. ^*p* > 0.05; *p < 0.05; ^**^p < 0.01; ^***^p < 0.001. Error bars show the standard deviation.

Our results showed that gramineous biomass proportion variation across the three alpine ecosystems explained 99% of the WUEe change (Figure 7B, *p* < 0.001). Besides, Cyperaceae biomass proportion changes showed a significant positive relationship with WUEe across the three alpine ecosystems. Unlike WUEe, no significant relationships were detected between community biomass characteristics and WUEc. However, community species diversity exhibited strong correlations with WUEc, the species richness change among the alpine ecosystems was negatively related to WUEc (Figure 7C, *p* < 0.01), and the *H*′ change explained 99% of the WUEc variation (Figure 7D, *p* < 0.001).

## Discussion

### Comparison of the Ecosystem Water Use Efficiency and Canopy Water Use Efficiency in the Three Alpine Ecosystems

Water use efficiency has been intensively analyzed at the leaf scale through the chamber and isotopic methods and at regional scales through modeling within remotely sensed data (Béziat et al., 2013; Kool et al., 2014; Liu W. et al., 2021). In this study, the overall mean observed that WUEe varied relatively lesser among the three alpine ecosystems and between years (Figure 4C; Table 2). The mean daily WUEe in alpine steppe and alpine meadow is slightly higher than the results obtained in a dry alpine steppe (0.31–1.38 g C kg^–1^ H_2_O) by Hu et al. (2008), when compared with the growing season monthly WUEe (0.03–1.64 g C kg^–1^ H_2_O) of FLUXNET sites (Law et al., 2008). The yearly WUEe of the alpine swamp was compared with the WUEe in peak growing season of various land use of temperate steppe of Inner Mongolia (more than 2 g C kg^–1^ H_2_O) (Niu et al., 2011; Li et al., 2016), which indicates that alpine swamp had a higher ecosystem WUE, perhaps implying that increased water availability may improve the WUEe in alpine ecosystems. This could be attributed to the GPP increase response to water availability being more sensitive than the response to ET, which was also concluded in a temperate grassland manipulative experiment (Niu et al., 2011).

**TABLE 2:** Sum of daily GPP, ET, T, and LAI during each growing season and annual WUE at the three Qinghai–Tibetan Plateau ecosystems.

Site	Year	Precipitation (mm)	LAI sum	GPP (gC m^–2^)	ET (kg H_2_O m^–2^)	T (kg H_2_O m^–2^)	WUEe (gC kg^–1^ H_2_O)	WUEc (gC kg^–1^ H_2_O)
Alpine steppe	2016	388.73	67.13	320.54	233.60	38.35	1.37	8.36
	2017	383.62	74.85	379.57	254.75	37.35	1.49	10.16
	2018	530.17	83.04	369.83	275.35	44.68	1.34	8.28
Alpine meadow	2017	627.97	294.79	812.94	549.55	337.48	1.48	2.41
	2018	629.19	287.94	866.13	481.12	345.01	1.80	2.51
Alpine swamp	2017	596.12	239.30	1289.71	596.21	248.48	2.16	5.19
	2018	614.60	234.31	1240.47	574.28	239.44	2.16	5.18

The variation range of overall mean daily WUEc of the three alpine ecosystems was larger than that of WUEe (Figure 4D) and, unlike WUEe, the WUEc values of the three sites were significantly different from each other. However, the lower site of alpine meadow (Figure 4C) was compared with that of temperate steppe (2.1–4.6 g C kg^–1^ H_2_O) (Niu et al., 2011); WUEc of alpine steppe (Figure 4D) was nearly two times higher than other reported temperate ecosystems and was even higher than the monthly results of peak growing season (4.9 g C kg^–1^ H_2_O) (Li et al., 2016). Besides, the moisture limits, which commonly occurred in the arid or semi-arid steppe area, accordingly caused ET suppression (Cienciala et al., 1994; Zhu et al., 2016). This may also be attributed to the high elevation of these two ecosystems (both higher than 4,000 m; Table 1), which are accompanied by strong photosynthetically active radiation at a low temperature (Figure 1). Correspondingly, the improved GPP and suppressed T jointly promoted WUEc.

### Comparison of the Regulation Mechanism Between Seasonal Variation of Ecosystem Water Use Efficiency and Canopy Water Use Efficiency

Few studies have examined the alpine ecosystem WUE response to changing environmental factors, especially for WUEc. It is generally understood that control of WUE varies with the scale of analysis (Hu et al., 2008; Niu et al., 2011). Thereby, limited understanding of the mechanisms controlling WUEc makes it more difficult to help Tibetan people to mitigate and adapt to the rapid regional change. As the data and analysis showed in this research, the LAI increases were significantly positively related to WUEe at all three sites (Figure 6D, *p* < 0.001), which is consistent with a temperate case study (Hu et al., 2008). However, the T/ET, which is commonly positively related to LAI change, was assumed to have a similar relationship with WUEe. This is the case for alpine meadow and swamp (Figure 6A, *p* < 0.001), but the positive relationship was not significant in the alpine steppe, which may be caused by T/ET coupling variation and the metrological and climatological disasters that frequently occur in the alpine steppe area, resulting in severe disturbance to WUE. The T/ET and LAI consistently showed exponential relationships with WUEc (Figures 6C,D), which suggests that the higher WUEc occurred when the plant began to grow. At this early growth stage, the photosynthetic conditions show younger leaf tissue with more active enzymes (Nath et al., 2013; Panda and Sarkar, 2013), and the leaves generally do not shade each other. As the plants gradually grew toward the maximum leaf coverage limitation for each community, the light competition and shading effect increased in the community, and consequently, the GPP growth would not catch up with the T growth and the rapidly decreased WUEc of early spring showed a gentler decrease (Figure 6C). This would then present as a WUEc decrease to a stable lower level when LAI had sufficiently increased (Figure 6D).

### Effect of Community Traits on the Inter-Annual and Inter-Ecosystem Variation in Ecosystem Water Use Efficiency and Canopy Water Use Efficiency

Leaf area index, T/ET, and the components of ET are common and critical regulation factors of WUEe (Hu et al., 2008; Li et al., 2016). The community characteristics, including species composition and diversity, have been reported to be closely related to LAI or carbon and vapor exchange (Dong et al., 2015; Li et al., 2015; Wei et al., 2015). The plant community is usually constituted with many coexisting species that may differ in water use strategy (Niu et al., 2011; Ens et al., 2015), thus appearing as considerable differences in WUE in leaf scale of species (Cernusak et al., 2010). The water use characteristics of various species that grow in the Qinghai–Tibetan Plateau were especially diversiform (Wang et al., 2004) because of their great habitat diversity. Proper plant functional group dividing methods would unexpectedly explain WUE variation across ecosystems (Figures 7A, B). The results shown in Figure 7B support this hypothesis as the gramineous biomass proportion was significantly negatively related to WUEe (*p* < 0.001). This may be attributed to the stronger drought-endurance of gramineous species (Panda and Sarkar, 2013; Hamilton et al., 2015; Zhang et al., 2017). A higher gramineous biomass proportion may, therefore, suggest a dry environment, which implied a lower WUEe and T/ET.

The species diversity indices were commonly negatively correlated with WUEc across alpine ecosystems (Figures 7C,D). We supposed that, in the community with complex synusia, the species with higher synusia occupy the best light conditions (Zhang L. et al., 2014), and usually obtained the higher WUE at the leaf scale, thus the species in lower synusia could only use the residue light resources. In a non-water limited situation, because abundant resources compensate for a lack of resources (Lassaletta et al., 2014), the GPP of lower synusia would decrease more than T, thereby the lower WUEc of these supplementary species in low synusia would suppress the WUE of the entire canopy. In contrast, in dry or other extreme environments, the community could only maintain simple synusia and few dominant species, and the vegetation cover would be too low to cause light competition between species, which may explain the high WUEc in barren ecosystems such as alpine steppe.

### Limitation and Outlook

There were three observation sites of typical ecosystems in this unique high-altitude region involved in this research, which limited the accurate understanding and creditable analysis across varied ecosystems within large spatial differences. For instance, the positive correlation trends observed between WUEe and LAI (Figure 6B) might be subject to amendment, if more different ecosystems had been included in the analysis, since LAI of more humid ecosystems might largely exceed the present maxim LAI recorded in the alpine meadow. Since the linear positive relationship probably has a threshold value, it could bend the trend. Besides, there is still uncertainty in obtaining the accurate daytime Re (Tcherkez et al., 2017), and the approach applied in our research has been argued to overestimate GPP in temperate forests (Wehr et al., 2016), which might hamper the accurate calculation of WUE.

Our findings were concentrated on revealing the regulation between community composition and structure with WUE in ecosystem and canopy scale, which could promote the understanding and accurate access to WUE modified by human activities and climate-driven community change. However, they lack an insightful understanding of the physiological mechanisms that drive the variation in WUEc across these varied alpine ecosystems. In addition, it should be stressed out that our observed phenomenon (e.g., Figure 7B) might only be applied to assess WUE in a limited area (alpine grasslands of Tibetan Plateau) or other grasslands dominated by gramineous and Cyperaceae plants, where the ecosystems commonly consist of two clearly divided function groups that largely diverge in water use strategy.

In our mind, a crucial direction for future progress will be used to determine the accurate regulation trends between community composition and structure and WUE, which covers more humid and arid ecosystems and observation sites distributed on the Qinghai–Tibetan plateau. With a deeper understanding of the suitable applied spatial range to these trends, the question of how to obtain a more accurate WUE variation trend and its suitable applied spatial range should be rephrased as follows: Does the composition and structure change across spatial distance or time series present a consistent regulation? Can the obtained trends be successfully applied to a more extreme environment and be mechanically explained by physiological factors? Once the mechanistic scaling of the community composition and structure effects on WUE response to human activities and climate change is successfully established, such a model would inform dynamic Earth-system models to calculate the effects of changing community on ecosystem atmosphere carbon and water exchange, which in turn promotes the accurate prediction on future water and carbon balance of the Qinghai–Tibetan Plateau.

## Conclusion

Combining the continuously observed eddy covariance system data with the ET partitioning ability of the S-W model, this study was divided into specific components of ecohydrological processes in the three typical alpine ecosystems. Our results showed that the mean yearly GPP was 356.65, 839.54, and 1265.09 g C m^–2^ in the alpine steppe, alpine meadow, and alpine swamp ecosystems, respectively; the mean yearly ET was 254.57, 515.34, and 585.25 kg H_2_O m^–2^ in the three typical ecosystems, respectively; the yearly WUEe was 1.40, 1.63, and 2.16 g C kg^–1^ H_2_O, in the three typical ecosystems, respectively, and the yearly WUEc was 8.93, 2.46, and 5.19 g C kg^–1^ H_2_O in the three typical ecosystems, respectively. A nonlinear effect in WUEc was regulated by LAI and T/ET in alpine ecosystems. Our results also imply that changes in community composition and structure across alpine ecosystems may have a primary impact on WUEc. Our findings indicate that there is a fundamental divergence in driving factors and regulation mechanisms between WUEe and WUEc in the alpine ecosystems.

## Data Availability Statement

The original contributions presented in the study are included in the article/supplementary material, further inquiries can be directed to the corresponding authors.

## Author Contributions

YL and ZH provided the idea for this research and prepared the manuscript. YL and QS contributed to the experimental design. YL and XZ collected the data in the study site. ZC and XZ contributed to the data analysis and figure-making. JD and YZ contributed to the revision of this manuscript. All authors contributed to the article and approved the submitted version.

## Conflict of Interest

The authors declare that the research was conducted in the absence of any commercial or financial relationships that could be construed as a potential conflict of interest.

## Publisher’s Note

All claims expressed in this article are solely those of the authors and do not necessarily represent those of their affiliated organizations, or those of the publisher, the editors and the reviewers. Any product that may be evaluated in this article, or claim that may be made by its manufacturer, is not guaranteed or endorsed by the publisher.

## Footnotes

1
https://ladsweb.modaps.eosdis.nasa.gov/
